# Adiponectin DNA methylation in South African women with gestational diabetes mellitus: Effects of HIV infection

**DOI:** 10.1371/journal.pone.0248694

**Published:** 2021-03-22

**Authors:** Stephanie Dias, Sumaiya Adam, Yoonus Abrahams, Paul Rheeder, Carmen Pheiffer

**Affiliations:** 1 Biomedical Research and Innovation Platform (BRIP), South African Medical Research Council, Tygerberg, Cape Town, South Africa; 2 Department of Obstetrics and Gynecology, University of Pretoria, Pretoria, South Africa; 3 Division of Medical Physiology, Faculty of Health Sciences, Stellenbosch University, Tygerberg, Cape Town, South Africa; 4 Department of Internal Medicine, Faculty of Health Sciences, University of Pretoria, Pretoria, South Africa; Texas A&M University College Station, UNITED STATES

## Abstract

DNA methylation is increasingly recognized as a potential biomarker of metabolic disease. However, there is limited information on the impact of human immunodeficiency virus (HIV) infection on the candidacy of DNA methylation to serve as molecular biomarkers. This study investigated the effect of HIV infection on DNA methylation patterns in the peripheral blood of South African women with (n = 95) or without (n = 191) gestational diabetes mellitus (GDM). DNA methylation levels at eight CpG sites in the adiponectin gene *(ADIPOQ)* promoter were measured using bisulfite conversion and pyrosequencing. Differences between HIV negative (^-^) and positive (^+^) women were observed. In HIV^-^ women, methylation at CpG -3400 was lower in GDM^+^ women compared to those with normoglycemia (8.5-fold; p = 0.004), and was associated with higher fasting glucose (β-co-efficient = 0.973; p = 0.006) and lower adiponectin (β-co-efficient = -0.057; p = 0.014) concentrations. These associations were not observed in HIV^+^ women. *In silico* analysis showed that Transcription Factor AP2-alpha is able to bind to the altered CpG site, suggesting that CpG -3400 may play a functional role in the regulation of *ADIPOQ* expression. Our findings show that DNA methylation differs by HIV status, suggesting that HIV infection needs to be taken into consideration in studies exploring DNA methylation as a biomarker of GDM in high HIV prevalence settings.

## Introduction

Gestational diabetes mellitus (GDM) is defined as glucose intolerance that is first diagnosed during pregnancy, with normal glucose tolerance usually restored after delivery [1]. GDM affects approximately 14% of pregnant women globally [2]. Without appropriate diagnosis and management, GDM is associated with adverse short-and long-term pregnancy outcomes [3, 4]. Perinatal complications include caesarean delivery, preeclampsia, macrosomia, birth injury, neonatal hypoglycaemia and postpartum haemorrhage [4, 5]. Moreover, both mothers and their offspring have an increased risk of developing type 2 diabetes (T2D) and obesity in later life [6–9]. GDM is diagnosed using the oral glucose tolerance test (OGTT) at 24–28 weeks of gestation [10]. Although universal screening with the OGTT is recommended for all pregnant women [11], many countries use a two stage risk factor-based selective screening strategy due to costs and ease [12–15]. As such, only women with traditional risk factors for GDM (advanced maternal age, obesity, family history of diabetes mellitus, delivery of a previous baby more than four kilograms, glucosuria, previous recurrent pregnancy loss, stillbirth, or birth of a baby with congenital abnormalities) [12, 16] are referred for OGTT screening. Due to the poor predictive value of traditional risk factors in identifying women with GDM and the challenges of the OGTT, which include the requirement for fasting and multiple blood draws, diagnosis of GDM is suboptimal. Accordingly, increased efforts are being directed towards the identification of sensitive and specific biomarkers to detect GDM. Such biomarkers could be useful to facilitate early risk stratification and intervention strategies to better manage GDM and improve pregnancy outcomes.

Epigenetics reflect the interplay between gene-environment interactions [17, 18], and are increasingly being implicated in the pathophysiology of metabolic diseases, including GDM. More recently, epigenetic mechanisms have attracted considerable interest as diagnostic or prognostic biomarkers of disease. DNA methylation, the most widely studied and best characterized epigenetic mechanism, refers to the addition of a methyl group to the fifth carbon position of a cytosine residue within a cytosine-phosphate-guanine (CpG) dinucleotide. This modification regulates the transcriptional potential of the genome and is known to affect gene expression pathways associated with a range of pathophysiological processes including glucose homeostasis, insulin signalling, inflammation and adipogenesis [19–21]. In recent years, accumulating evidence show that aberrant DNA methylation patterns in tissue are reflected in peripheral blood, which has sparked interest in its potential to serve as biomarkers of disease [22–24]. As such, a number of studies have reported altered gene-specific methylation during GDM [25–28].

Adiponectin is an adipose tissue-derived hormone that modulates whole-body energy homeostasis by regulating glucose and lipid metabolism [29, 30]. In metabolic tissues, adiponectin enhances insulin sensitivity by promoting glucose utilization and fatty acid oxidation. Adiponectin serum levels are negatively correlated with obesity and obesity-related metabolic diseases such as insulin resistance, T2D and cardiovascular disease [31–33]. In recent years, the role of adiponectin during pregnancy has attracted interest, particularly due to evidence that adiponectin may be secreted from the placenta [34]. Adiponectin concentrations progressively decline with increasing insulin resistance during pregnancy [35, 36], and low adiponectin levels during early pregnancy is associated with the development of GDM [37]. The potential of maternal serum adiponectin concentration to serve as a biomarker of GDM is widely reported [38–42]. Altered DNA methylation of the adiponectin gene has been identified as a key mechanism that regulates serum adiponectin expression, and to influence glucose and lipid metabolism [43].

Human immunodeficiency virus (HIV) infection alters epigenetic mechanisms such as DNA methylation [44, 45], which may affect their ability to serve as biomarkers of GDM. Sub-Saharan Africa accounts for 68% of the global HIV burden, with an estimated 25.7 million people infected with HIV [46]. Given the high prevalence of HIV in Africa, particularly in women of reproductive age [47], it is important to examine the effect of HIV infection on the potential of DNA methylation to serve as a biomarker for GDM. Previous studies investigating DNA methylation during GDM were conducted in HIV negative (HIV^-^) women [25] or in low HIV prevalence settings without consideration of HIV status [48, 49]. This study aimed to address the scarcity of studies exploring the interaction between HIV and DNA methylation. We measured DNA methylation levels at eight CpGs at -3413, -3410, -3400, -3372, -473, -415, -112 and -45bp upstream of the *ADIPOQ* promoter in the peripheral blood of HIV infected and uninfected South African pregnant women with (n = 95) or without (n = 191) GDM using pyrosequencing. Associations between altered CpG methylation and clinical characteristics were examined. The functional significance of altered CpGs were explored using *in silico* transcription factor binding prediction.

## Materials and methods

### Study population

**Fig 1:**
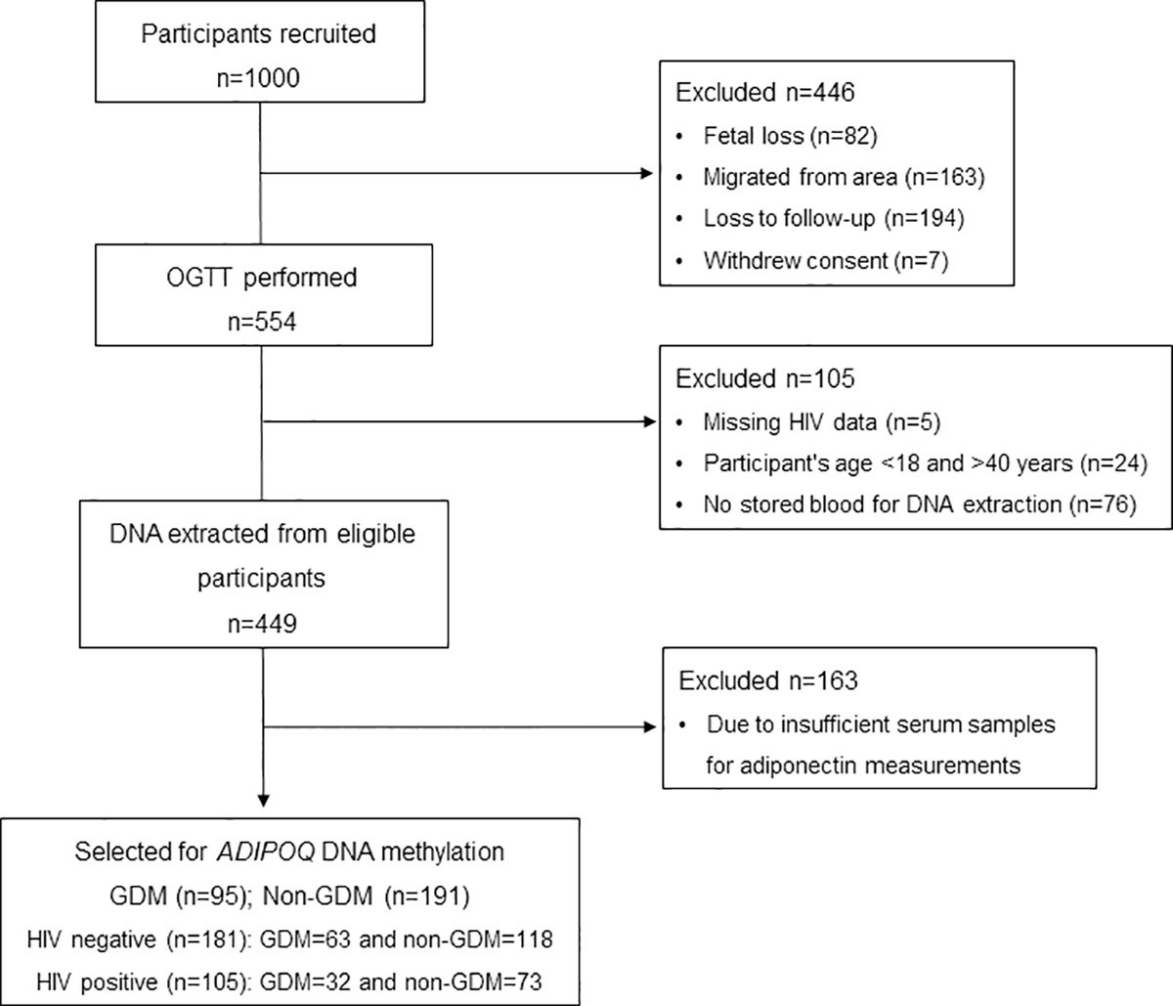
Flow diagram for study participants. A subset of women with (n = 95) and without (n = 191) GDM were selected for this cross-sectional study and were stratified according to HIV negative and HIV positive groups. HIV: Human Immunodeficiency Virus.

This research forms part of a larger study investigating screening strategies for GDM in a South African population [12]. One thousand pregnant black African women attending a primary care clinic in Johannesburg, South Africa were recruited between September 2013 to March 2016. Women with singleton pregnancies, and who did not have pre-existing diabetes (type 1 diabetes (T1D) and T2D) were included in the study. Participant selection for this study is shown in Fig 1. In total 95 women with GDM and 191 women without GDM were included in this study. Women were stratified according to their GDM (GDM (GDM ^+^); no GDM (GDM^-^)) and HIV (HIV positive (HIV^+^); HIV negative (HIV^-^)) status as follows: GDM^-^ HIV^-^: n = 118, GDM^+^ HIV^-^: n = 63 and GDM^-^ HIV^+^: n = 32, GDM^+^ HIV^+^: n = 73. Of the 105 HIV^+^ women, 36 were on anti-retroviral treatment (ART) (GDM^+^ (n = 12) and GDM^-^ (n = 24)), 68 were ART naïve (GDM^+^: n = 20, GDM^-^: n = 48) and one had missing data, which was not included in the ART analysis. At recruitment, demographic data and risk factors for GDM, i.e. advanced maternal age (age > 35 years), obesity (body mass index (BMI) > 30 kg/m^2^), family history of diabetes mellitus, delivery of a previous baby more than four kilograms, glucosuria, previous recurrent pregnancy loss, stillbirth, or birth of a baby with congenital abnormalities, were assessed using a standardised questionnaire (S1 Questionnaire). Anthropometric measurements including age, height (cm) and weight (kg) were obtained using standard procedures as previously described [12], and BMI was calculated as weight (kg)/height squared (m^2^). At their first visit, random glucose and glycated haemoglobin (HbA1c) concentrations were measured using a glucometer (Roche Diagnostics, Mannheim, Germany) and the point-of-care Afinion system (Alere Technologies, Oslo, Norway), respectively. All women with random glucose and HbA1c concentrations more than 11.1 mmol/L 6.5%, respectively, were excluded. Women included in the study were requested to return within two weeks in a fasted state for blood collection. Ethical approval for this study was granted by the University of Pretoria Health Sciences Ethics Committee (180/2012). The study was conducted according to the Declaration of Helsinki and all women gave written informed voluntary consent after the procedures had been fully explained in the language of their choice [12].

### Study procedures

Study procedures were described previously [12]. GDM was diagnosed using the 75-g 2-hr oral glucose tolerance test (OGTT) at 24–28 weeks of pregnancy according to the International Diabetes and Pregnancy Study Group (IADPSG) criteria, and diagnosed if at least one glucose value was met (fasting plasma glucose ≥ 5.1mmol/L, 1 hr OGTT ≥ 10 mmol/L or 2 hr OGTT ≥ 8.5 mmol/L) [50]. HIV testing was offered to all pregnant women using rapid HIV kits and results were confirmed with a different kit according to the guidelines of the South African Department of Health [51]. HIV positive women were treated with Atripla™; a fixed-dose coformulation of three anti-HIV drugs, efavirenz, emtricitabine and tenofovir given once-daily [51]. After an overnight fast, blood samples were collected for measuring fasting glucose concentrations (Vermaak and Partners, Pretoria, South Africa) or stored at -80 ^o^C to assess serum adiponectin concentrations (human adiponectin enzyme-linked immunosorbent assay (ELISA) (Merck, Dermstadt, Germany)) and profile DNA methylation.

### DNA methylation analysis

Genomic DNA was extracted from 2 ml of whole blood in ethylenediaminetetraacetic acid (EDTA) tubes using the QIAamp DNA Blood Midi Kit (Qiagen, Hilden, Germany). DNA concentration was measured using the Qubit Flourometer (Invitrogen, Carlsbad, USA) with the Quanti-iT dsDNA Broad Range assay kit (ThermoFisher, Massachusetts, USA), according to the manufacturer’s instructions. DNA methylation analysis was performed using pyrosequencing. Briefly, 500 ng of DNA was bisulfite converted using the EpiTech Fast DNA Bisulfite Kit (Qiagen, Hilden, Germany), according to the manufacturer’s instructions. PCR was performed on 20 ng of bisulfite converted DNA using the Pyromark PCR kit and pyrosequencing was conducted using the PyroMark Gold Q96 reagent kit and the PyroMark Q96 pyrosequencer (Qiagen). Bisulfite treatment and pyrosequencing assays were tested using bisulfite conversion controls and methylated standards (0% to 100%) (S1 Fig) (Qiagen), according to manufacturer’s instructions. Samples were randomly selected and tested in duplicate to confirm reproducibility of methylation data.

### Primers

The three primer sets used for DNA methylation analysis were selected from publications that identified regions important for *ADIPOQ* gene regulation [49, 52] or designed using the PyroMark Assay Design Software (version 2.0.2.5, Qiagen). Primer set 1 (R1) represents 4 CpGs at -3413, -3410, -3400 and -3372 (region C in Bouchard et al. 2012 [49]) and primer set 2 (R3) represents 2 CpGs at -112 and -45 (Houshmand-Oeregaard et al. 2017 [52]) upstream from the transcription start site (TSS). Primer set 3 (R2) (2 CpGs at -473 and -415 upstream from the TSS) was designed by us. Primer sets included a forward, reverse and sequencing primer (Integrated DNA Technologies, Inc., South Africa). The primer and target sequences, chromosomal location and amplicon lengths are listed in S1 Table.

### In silico analysis

*In silico* analysis was conducted to identify potential transcription factors that bind to regions of altered CpG methylation. Transcription factor prediction software, ALIBABA 2.1 [53] and ALGGEN-PROMO [54], was used to identify putative transcription factors with binding sites overlapping CpG -3400 in the region between -3425 bp to -3383 bp (42 bp) within the human *ADIPOQ* gene sequence (GRCh38/hg38) (https://www.ensembl.org/index.html). Transcription factor predictions were cross-referenced using the MeDReader database [55] to assess the likelihood of these transcription factors binding to highly methylated regions.

### Statistical analysis

Statistical analysis was conducted using STATA 14 (StataCorp, College Station, USA). Data are expressed as the median and interquartile range (25^th^– 75^th^ percentile) and categorical data are expressed as count (percentage). Data normality was tested using the Shapiro-Wilk test. Numerical data were analyzed using the Mann-Whitney test, while categorical variables were analyzed using the Chi-squared test. Associations between GDM and differentially methylated CpGs were analysed using logistic regression adjusting for age, BMI and gestational age. GDM was classified as the binary dependent variable and DNA methylation as the continuous independent variable. Associations between DNA methylation and clinical characteristics were assessed using univariable and multivariable linear regression analysis adjusting for age, BMI and gestational age. Graphs were drawn in Prism 7, Version 7.03 (GraphPad, La Jolla, USA). A DNA methylation cut-off of >1.5-fold and a p-value of ≤0.05 (α) was considered statistically significant. The p-values were adjusted for multiple testing using Bonferroni correction cut-off at α/n (n = number of CpG sites).

## Results

### Participants characteristics

Participant characteristics are presented in Table 1. All measures of glucose concentration were higher in HIV^-^ women with GDM compared to those with normoglycemia. In HIV^+^ women, fasting and OGTT glucose concentrations were higher in women with GDM compared to those with normoglycemia, while no differences in HbA1c levels were observed. HIV^-^ women with GDM had more risk factors and lower serum adiponectin concentrations than women without GDM. These GDM associated differences in risk factors and adiponectin concentrations were not observed in HIV^+^ women. No difference in the percentage of women on ART was observed between GDM and non-GDM groups. The majority of women with GDM (80/95; 84.2%) were diagnosed on fasting plasma glucose alone, whilst 2/95 (2.1%) were diagnosed on 2 hr OGTT alone, and a further 13/95 (13.7%) were diagnosed with fasting glucose plus one or two additional abnormal glucose reading (S2 Table).

**Table 1:** Participant characteristics.

Participant Characteristics	HIV^-^			HIV^+^		
	GDM^-^	GDM^+^	p-value	GDM^-^	GDM^+^	p-value
Participants: n	118	63		73	32	
Age (years)	26.0 (23.0–30.0)	28.0 (24.0–32.0)	0.079	29.0 (25.0–33.0)	30.0 (25.0–32.0)	0.613
BMI (kg/m^2^)	25.6 (22.7–28.6)	26.9 (22.9–30.7)	0.232	25.8 (22.3–29.7)	26.6 (24.4–33.6)	0.085
Gestational Age (weeks)	25.0 (20.0–28.0)	25.0 (21.0–27.0)	0.754	26.0 (22.0–28.0)	25.0 (20.5–26.5)	0.306
Random glucose (mmol/L)	4.3 (3.9–4.7)	4.6 (4.1–5.0)	<0.001	4.4 (4.1–4.9)	4.6 (4.3–5.2)	0.124
Fasting glucose (mmol/L)	4.3 (4.0–4.6)	5.7 (5.3–6.0)	<0.001	4.5 (4.2–4.8)	5.4 (5.3–5.7)	<0.001
OGTT 1 hr (mmol/L)	5.3 (4.5–6.3)	6.3 (5.6–8.3)	<0.001	5.5 (4.7–6.3)	5.9 (5.3–7.0)	0.029
OGTT 2 hr (mmol/L)	5.2 (4.6–5.7)	6.1 (5.1–7.3)	<0.001	5.1 (4.3–5.8)	6.1 (5.1–7.1)	<0.001
HbA1c (%)	5.1 (4.8–5.3)	5.3 (5.0–5.5)	0.007	5.3 (5.1–5.5)	5.3 (5.1–5.5)	0.886
Adiponectin (μg/ml)	9.7 (7.3–14.5)	7.6 (4.9–11.8)	0.009	14.4 (9.4–20.3)	14.0 (7.2–19.6)	0.427
^a^Risk Factors: n (%)			0.038			0.143
None	75 (63.6)	30 (37.6)		34 (46.6)	10 (31.3)	
≥ 1 risk factor	43 (36.4)	33 (52.4)		39 (53.4)	22 (68.7)	
ART: n (%)	-	-	-			0.567
Yes				24 (32.8)	12 (38.7)	
No				49 (67.1)	19 (61.3)	

^a^Risk factors: advanced maternal age (age > 35 years), obesity (BMI > 30 kg/m^2^), family history of diabetes mellitus, delivery of a previous baby more than four kilograms, glucosuria, previous recurrent pregnancy loss, stillbirth, or birth of a baby with congenital abnormalities. Data are expressed as the median and interquartile range (25th–75th percentiles) or as count (percentage). ART: antiretroviral treatment; BMI: body mass index; OGTT: oral glucose tolerance test; HbA1c: glycated haemoglobin.

### HIV infection alters the association between GDM and *ADIPOQ* DNA methylation

**Fig 2:**
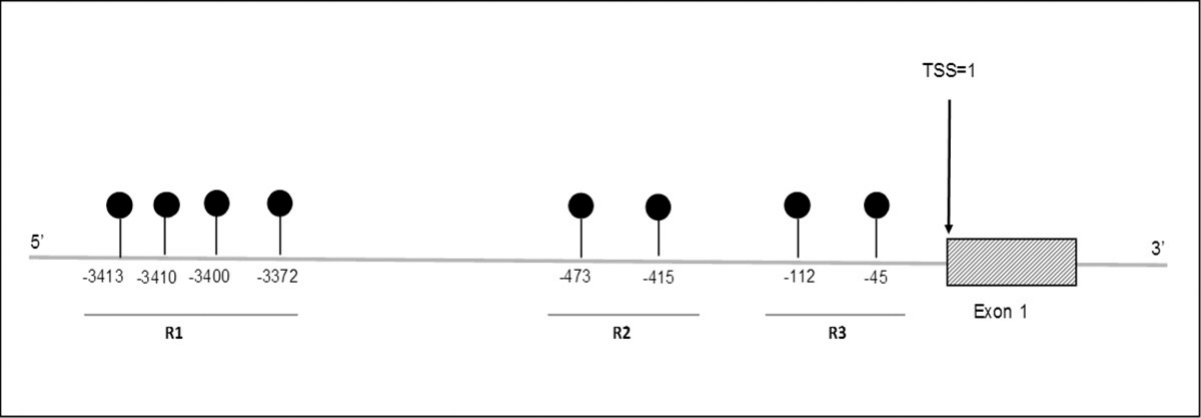
Schematic illustration of the location of the eight CpG sites upstream of the *ADIPOQ* gene. Region 1 (R1) represents 4 CpGs at -3413, -3410, -3400 and -3372, R2 represents 2 CpGs at -473 and -415 and R3 represents 2 CpGs at -112 and -45. TSS: Transcription start site.

**Fig 3:**
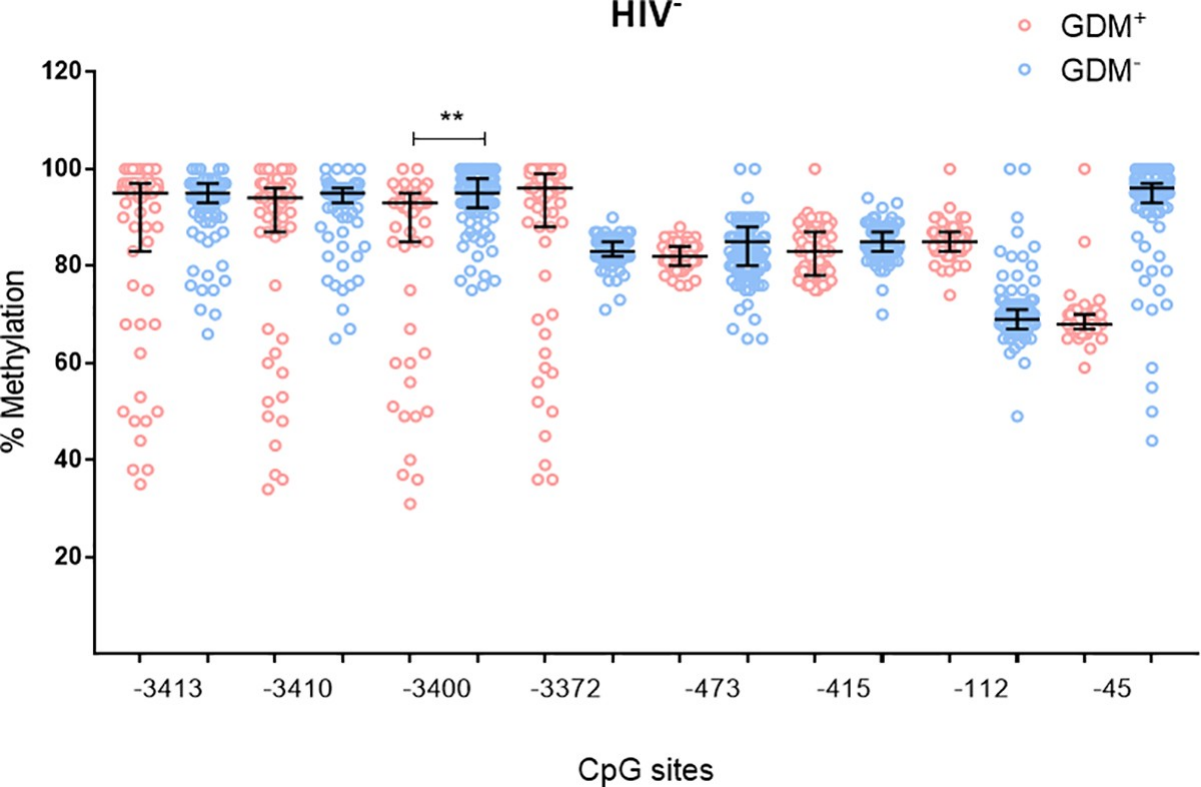
*ADIPOQ* promoter DNA methylation and GDM status in HIV^-^ women. DNA methylation at eight CpGs within the promoter of *ADIPOQ* was measured in HIV^-^ pregnant women with and without GDM (n = 118 GDM^-^; n = 63 GDM^+^). Data are represented in a scatter dot plot as the median and interquartile range. **p<0.01.

**Fig 4:**
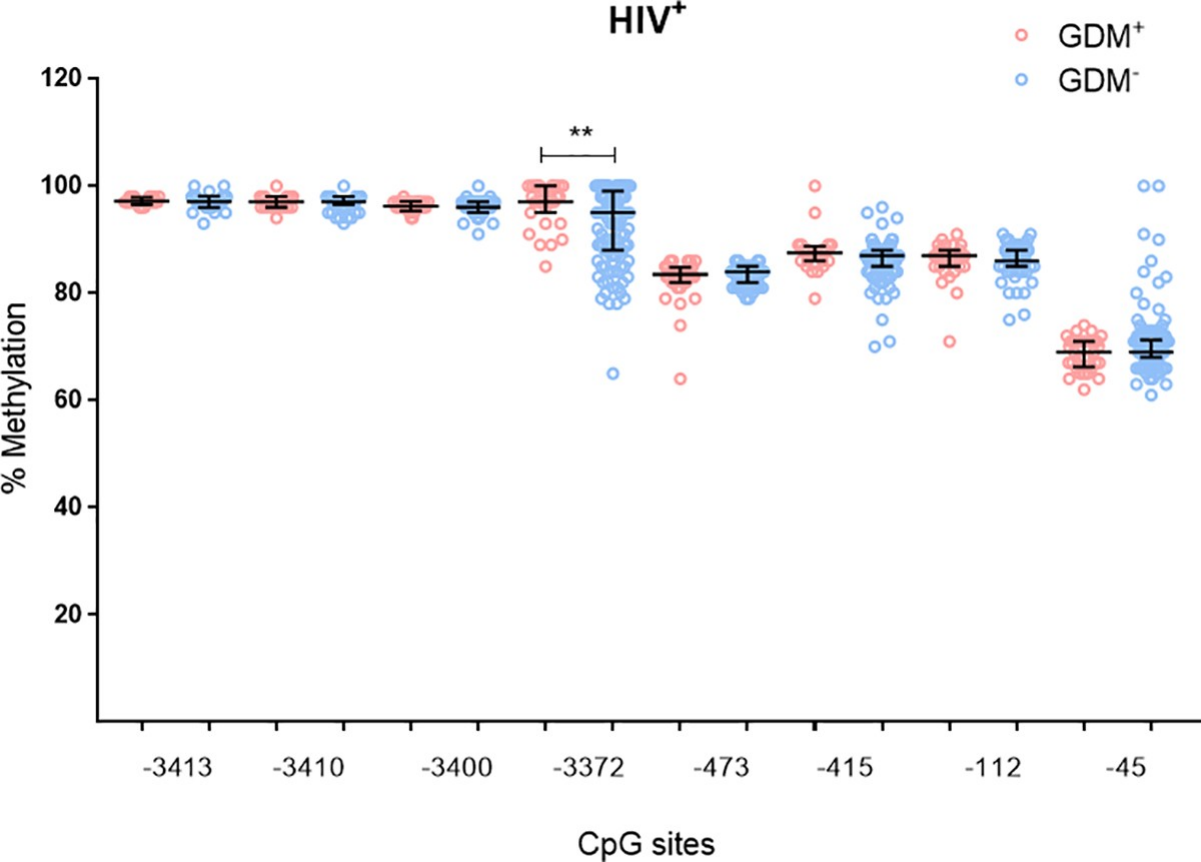
*ADIPOQ* promoter DNA methylation and GDM status in HIV^+^ women. DNA methylation at eight CpGs within the promoter of *ADIPOQ* was measured in HIV^+^ pregnant women with and without GDM (n = 73 GDM^-^; n = 32 GDM^+^). Data are represented in a scatter dot plot as the median and interquartile range. **p<0.01.

**Fig 5:**
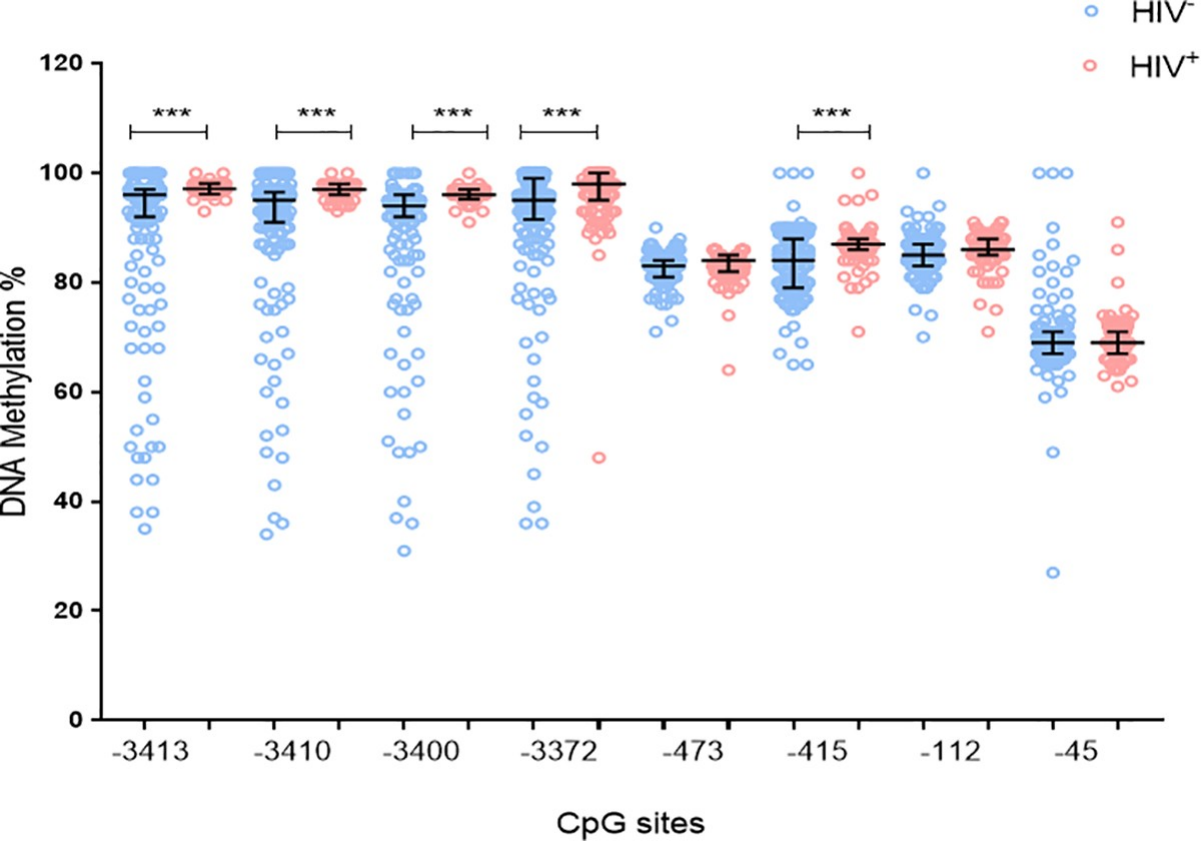
*ADIPOQ* promoter DNA methylation and HIV status. DNA methylation at eight CpGs within the promoter of *ADIPOQ* was measured in HIV^-^ (n = 181) and HIV^+^ (n = 105) pregnant women. Data are represented in a scatter dot plot as the median and interquartile range. ***p<0.001.

To investigate the effect of HIV infection on *ADIPOQ* DNA methylation levels during GDM, methylation levels at eight CpG sites within the *ADIPOQ* promoter were quantified. A schematic representation of the eight CpG sites in *ADIPOQ* promoter that were investigated are illustrated in Fig 2. Pyrosequencing showed high levels of methylation at all CpGs, with significant inter-individual heterogeneity observed between women (Figs 3 and 4). In HIV^-^ women, methylation at CpG -3400 was lower in GDM^+^ women compared to those with normoglycemia (8.5-fold; p = 0.004) (Fig 3). These differences were not observed in HIV^+^ women. In HIV^+^ women, methylation at CpG -3372 was higher in GDM^+^ women compared to GDM^-^ women (4.0-fold; p = 0.006) (Fig 4). Logistic regression adjusting for age, BMI and gestational age confirmed the association between GDM and DNA methylation at CpG -3400 in HIV^-^ women (β co-efficient = -3.954, 95% CI = -6.658 to -1.250, p = 0.004), while the association between GDM and DNA methylation at CpG -3372 in HIV^+^ women did not withstand adjusting for these factors (Table 2). Interestingly, lower methylation levels at five CpGs, -3413 (6.3-fold; p<0.001), -3410 (6.4-fold; p<0.001), -3400 (6.3-fold; p<0.001), -3372 (4.6-fold; p<0.001) and -415 (3.6-fold; p<0.001) were observed in HIV^-^ compared to HIV^+^ women (Fig 5). No differences in methylation levels at these CpGs were observed in HIV^+^ women on ART compared to ART naïve women (S2 Fig).

**Table 2:** Association between GDM and *ADIPOQ* DNA methylation levels in HIV^-^ and HIV^+^ women.

GDM status	β co-efficient	95% CI	p-value
***HIV***^***-***^			
CpG -3400	-3.954	-6.658; -1.250	0.004
***HIV***^***+***^			
CpG -3372	-1.693	-5.093; 1.708	0.329

Multivariable logistic regression analysis: Adjusted for age, body mass index and gestational age; β: beta; CI: Confidence interval. Statistical significance is indicated by p<0.05.

### DNA methylation and clinical characteristics

Since CpG -3400 was associated with GDM after adjusting for age, BMI and gestational age in HIV^-^ women, we further explored the association between methylation at this CpG and clinical characteristics. Univariable and multivariable regression adjusting for age, BMI and gestational age showed that methylation levels at CpG -3400 were positively associated with fasting glucose (β co-efficient = 0.720, 95% CI = 0.091; 1.349, p = 0.025) and negatively associated with serum adiponectin (β co-efficient = -0.050, 95% CI = -0.096; -0.004, p = 0.035) concentrations in HIV^-^ women (Table 3).

**Table 3:** Association between methylation at CpG -3400 and clinical characteristics in HIV^-^ women.

DNA methylation at CpG -3400	β	95% CI	p-value	β	95% CI	p-value
	Univariable			Multivariable		
Random glucose	-1.043	-50.509; 48.424	0.962	26.105	-56.978; 109.188	0.432
Fasting glucose	0.973	0.283; 1.662	0.006	0.720	0.091; 1.349	0.025
OGTT 1 hr	-0.061	-0.144; 0.022	0.150	-0.038	-0.116; 0.039	0.332
OGTT 2 hr	0.029	-0.089; 0.148	0.625	0.047	-0.066; 0.160	0.416
HbA1c	-0.0004	-0.001; 0.001	0.349	-0.0001	-0.001; 0.001	0.811
Adiponectin	-0.057	-0.102; -0.012	0.014	-0.050	-0.096; -0.004	0.035
Risk factors	-0.009	-0.066; 0.047	0.740	-0.013	-0.072; 0.045	0.658

Univariable linear regression analysis: DNA methylation and clinical parameters.

Multivariable linear regression analysis: Adjusted for age, body mass index and gestational age. β: beta coefficient; CI: Confidence interval. OGTT: oral glucose tolerance test; HbA1c: glycated haemoglobin; *ADIPOQ*: Adiponectin gene. p<0.05 indicate statistical significance.

### In silico analysis

**Fig 6:**
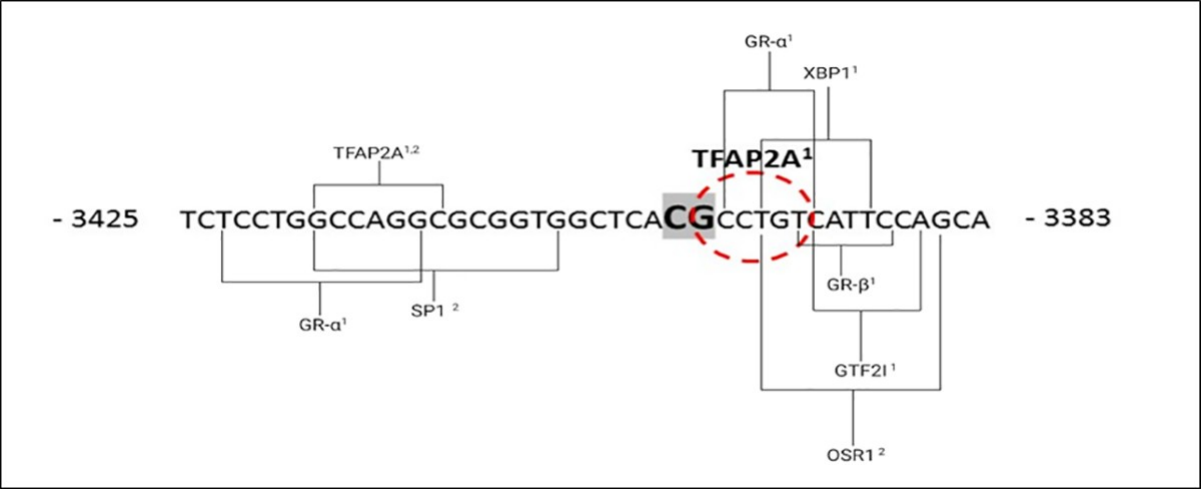
Schematic illustration of transcription factors binding to differentially methylated CpG -3400 and to adjacent regions. *In silico* transcription factor prediction analysis identified transcription factor AP2-alpha (TFAP2A) binding site directly over CpG –3400 upstream of the *ADIPOQ* gene transcription start site, using ALGGEN-PROMO^1^ and ALIBABA 2.1^2^ software. Eight other transcription factor binding sites were identified within the region -3425 to -3383 adjacent to the region of altered DNA methylation. The following factors were identified: *GRα*—Glucocorticoid Receptor Alpha; *XBP1—*X-Box Binding Protein 1; *SP-1—*Specificity Protein 1 Transcription Factor; *OSR1—*Odd-Skipped Related Transcription Factor 1; *GRβ*—Glucocorticoid Receptor Beta*; GTF2I* –General Transcription Factor IIi and *TFAP2A* - Transcription Factor AP2-alpha; Bold-face/highlighted nucleotides—indicate differentially methylated CpG -3400; Brackets—transcription factor binding regions adjacent to CpG -3400; Red dotted circle–TFAP2A binding site.

DNA methylation at CpG -3400 was inversely correlated with serum adiponectin concentrations in all HIV^-^ women, suggesting a role in gene regulation. Thus, to further explore the functional significance of CpG -3400 site, *in silico* transcription factor prediction was conducted to identify transcription factors capable of binding to altered methylation sites. A binding site for Transcription Factor AP2-alpha (*TFAP2A*) was identified directly over the altered methylated CpG -3400 site (Fig 6). It is known that DNA methylation is capable of interfering with transcription factor binding in adjacent regions and influencing gene expression. Thus, binding sites between -3425 bp to -3383 bp that spanned the investigated CpG -3400 were also explored. Transcription factor prediction software identified several other transcription factors: Glucocorticoid receptor alpha and beta (*GRα* and *GRβ*), X-Box Binding Protein 1 (*XBP1*), General Transcription Factor IIi (*GTF2I*), Specificity Protein 1 Transcription Factor (*SP-1*) and Odd-Skipped Related Transcription factor 1 (*OSR1*) capable of binding within the wider region spanning CpG -3400 site.

## Discussion

DNA methylation plays an important role in the pathophysiology of GDM and is widely explored as a potential biomarker that could facilitate risk stratification and intervention strategies to potentially better manage GDM and improve health outcomes. It is estimated that approximately 1.3 million pregnancies are complicated by HIV globally [46], with a large proportion of HIV infected women living in Sub-Saharan Africa [46]. Thus, it is important to examine the effect of HIV infection on DNA methylation, which may impact its candidacy to serve as a biomarker for GDM. However, studies on the interaction between HIV infection and DNA methylation are scant. Our findings provide novel evidence that HIV infection alters *ADIPOQ* DNA methylation during GDM. In HIV^-^ women only, methylation levels at CpG -3400 were lower in women with GDM compared to those with normoglycemia. Methylation levels at CpG -3400 were associated with higher fasting glucose and lower adiponectin concentrations in HIV^-^ women, but not in HIV^+^ women. *In silico* analysis demonstrated that transcription factor, *TFAP2A*, binds the differentially methylated CpG -3400, suggesting that it may play a role in regulating *ADIPOQ* expression.

Adiponectin is secreted by adipose tissue and plays a critical role in maintaining glucose homeostasis and insulin sensitivity [56]. Pregnancy is characterized by insulin resistance, which increases as pregnancy progresses in order to meet the nutritional demands of the growing fetus [57]. Accordingly, lower adiponectin concentrations are observed with pregnancy duration [35, 36]. Moreover, adiponectin levels are lower in women with GDM compared to pregnant women with normoglycemia [37], suggesting that adiponectin dysregulation plays a role in the pathophysiology of GDM. DNA methylation is an epigenetic mechanism that may regulate adiponectin expression [43]. Our findings are consistent with others who have similarly reported altered *ADIPOQ* methylation in populations from Denmark, Germany and French-Canadian Origin [48, 52, 58]. Ott et al. showed that the average *ADIPOQ* DNA methylation is higher in paired subcutaneous and visceral adipose tissue depots of German women with GDM compared to normoglycemia, suggesting that *ADIPOQ* DNA methylation may be involved in the complex metabolic and subclinical inflammatory milieu associated with insulin resistance and the risk of developing GDM. Furthermore, these authors showed that methylation levels at these CpG sites were correlated with lower *ADIPOQ* gene expression, supporting the functional relevance of these sites [48]. In a Danish population of adult offspring born to women with GDM, Houshmand-Oeregaard et al. reported increased *ADIPOQ* DNA methylation in subcutaneous adipose tissue, which was accompanied by decreased gene expression [52]. Moreover, studies have also reported altered *ADIPOQ* methylation in the placenta of pregnant women with obesity and GDM [58, 59]. These studies were conducted in low HIV prevalence settings and did not consider the effect of HIV on *ADIPOQ* DNA methylation.

Our findings are consistent with a previous study that similarly reported high CpG methylation in the *ADIPOQ* promoter. Ott et al. showed that methylation at highly methylated CpGs in the *ADIPOQ* gene were altered in peripheral blood of women with GDM compared to those with normoglycemia. Furthermore, these authors showed that although the overall correlation between DNA methylation levels in adipose tissue and maternal blood was low, hypermethylation of one specific CpG within *ADIPOQ* was conserved in visceral adipose tissue and maternal blood of women with GDM, supporting its biomarker potential [48]. In HIV^-^ women, DNA methylation at CpGs -3400 within the *ADIPOQ* promoter correlated with higher glucose and lower serum adiponectin concentrations, which is consistent with findings from Bouchard et al. who similarly reported an association between *ADIPOQ* methylation and hyperglycemia and circulating adiponectin concentrations during pregnancy [49]. Bouchard et al. quantified DNA methylation in the placenta at delivery, while DNA methylation in our study was measured in peripheral blood of women with an average gestational age of 23.6 weeks. These findings suggest that altered DNA methylation in the placenta at delivery are reflected in peripheral blood around 23 weeks of gestation, highlighting the potential of DNA methylation as a biomarker.

Although studies have previously reported on the effect of HIV infection on DNA methylation levels, these were not in the context of GDM [44, 60–63]. *In vitro* studies demonstrated that HIV infection modulates DNA methylation to promote viral integration into the host cell and to increase the virus’s ability to replicate, survive and establish latency [60, 61]. In our study, five CpG sites (-3413, -3410, -3400, 3373, and -415) were differentially methylated in HIV^-^ compared to HIV^+^ women, regardless of GDM status. Using the HumanMethylation450 Beadchip array, Zhang et al. similarly reported DNA methylation differences in HIV^-^ and HIV^+^ individuals [44]. A study investigating global DNA methylation in LINE-1 and AluYb8 repetitive elements showed lower global DNA methylation in infants exposed to HIV and ART *in utero* compared to unexposed infants [64]. In our study, *ADIPOQ* DNA methylation at CpGs -3413, -3410, -3400, 3373 or -415 did not differ in ART treated and ART naive HIV^+^ women. Moreover, our findings show that in contrast to HIV^-^ women, risk factors did not differ between HIV^+^ women with or without GDM, suggesting that screening for GDM using risk factors alone lacks sensitivity in HIV^+^ South African women, and may result in many HIV^+^ women with GDM remaining undiagnosed.

*ADIPOQ* DNA methylation was inversely correlated with serum adiponectin concentrations in HIV^-^ women, thus transcription factor, *TFAP2A*, predicted by *in silico* analysis to bind CpG -3400, may be involved in regulating *ADIPOQ* expression. *TFAP2A* forms part of the transcription factor activating protein 2 (*TFAP2)* family [65] that can directly transactivate their target genes by binding the same GC-rich consensus sequence [66, 67]. Studies have reported that *TFAP2* may play a role in regulating genes for insulin resistance and adiposity [68, 69]. Furthermore, *TFAP2A* has been shown to be widely expressed during embryonic development [65, 70], particularly in placenta where reduced expression of *TFAP2A* was identified in syncytiotrophoblast cells of pregnancies complicated by diabetes, hypertension and mild pre-eclampsia compared to age-matched controls, suggesting a link between *TFAP2A* expression and pregnancy outcomes [71]. These results suggest that altered methylation of CpG in the *ADIPOQ* promoter could potentially regulate *ADIPOQ* gene expression during GDM, which may differ in HIV^-^ and HIV^+^ pregnant women. Further mechanistic studies are required to elucidate the role of these putative transcription factors in regulating *ADIPOQ* expression during GDM.

To the best of our knowledge, this study is the first to explore the interaction between HIV infection and DNA methylation during GDM. A further strength of the study is the sample size (n = 286) which was larger than previous studies on DNA methylation and GDM and were adjusted for known confounders such as age, BMI and gestational age [48, 49]. We used pyrosequencing, which is considered the gold standard for DNA methylation analysis and is a highly reproducible method that is able to accurately detect small methylation differences [72]. However, we acknowledge that our study is not without limitations. All study participants are black South African pregnant females, and therefore generalizability to other populations may not be applicable. In addition, a large percentage of women were diagnosed with GDM using abnormal fasting glucose measurements only, which may have led to false positives due to non-adherence to fasting. However, these results should be interpreted with caution, since women with even mild GDM have showed improved maternal and neonatal outcomes with lifestyle intervention and medical management if necessary [73–75]. Furthermore, due to the cross-sectional nature of the study causality between HIV infection, *ADIPOQ* methylation and GDM cannot be determined, warranting further longitudinal studies. Peripheral blood consists of a variety of different cell types including erythrocytes, lymphocytes and platelets [76], which may confound methylation analysis. Although we previously reported no significant differences in cell type composition in a subset of our sample [25], we acknowledge the role of cell type composition as a possible confounder in our analysis. Furthermore, we did not quantify messenger RNA levels, thus we are not able to assess whether DNA methylation changes led to altered gene expression. We did however observe that *ADIPOQ* methylation at CpG -3400 was inversely correlated with serum adiponectin concentration levels, which is consistent with the role of DNA methylation to silence gene expression. Although DNA methylation is traditionally associated with gene silencing, increased methylation was associated with higher adiponectin expression in HIV^+^ women. These results support the view that DNA methylation is functionally complex and may regulate other mechanisms such as alternative splicing and microRNAs, and may even induce gene expression [77, 78]. Future mechanistic studies to explore the function of the observed methylation changes and the *in silico*-identified transcription factors are required. Importantly, DNA methylation is an epigenetic mechanism that is influenced by several environmental factors, and although we adjusted for age, BMI and gestational age, other factors such as diet, exercise and smoking status may have confounded our analysis [79–82]. Although, women were recruited from the same community and were likely to have similar environmental exposures, significant inter-individual heterogeneity was observed in our study, reflecting both genetic and environmental differences. Lastly, information on viral load, CD4^+^ count, immune status and HIV and ART duration, were not known, limiting our ability to fully understand the relationship between DNA methylation, GDM and HIV infection.

In conclusion, this study provides novel evidence that HIV infection alters the association between GDM and *ADIPOQ* DNA methylation in South African women. These findings have implications for biomarker discovery in high HIV prevalence settings and highlights the importance of considering HIV status in DNA methylation biomarker studies in such settings. Further studies are required to determine the functional significance of the DNA methylation changes observed in this study and to explore the relationship between DNA methylation, GDM and HIV infection.

## Supporting information

S1 Questionnaire Standardized clinic questionnaire amended for this study. (DOCX)

S1 Fig Primer sensitivity using known methylated standards (x-axis) for each pyrosequencing probe. The percentage (%) of methylation as determine (y-axis) for a) 4 CpGs (-3412, -3410, -3400, -3372) in R1, b) 2 CpGs (-473, -415) in R2 and c) 2 CpGs (-112, -45) in R3.
(DOCX)

S2 Fig DNA methylation levels in HIV+ women receiving antiretroviral therapy (ART) (n = 36) and those who were ART naïve (n = 69). DNA methylation levels at CpG -3413, -3410, -3400, -3372 and -415. *p<0.05.
(DOCX)

S1 Table Primer design for CpG sites in *ADIPOQ*. The target sequence, chromosomal location, target sequence and amplicon length used to design CpG assays.
(DOCX)

S2 Table Analysis of the International Association of Diabetes in Pregnancy Study Group Criteria in diagnosing GDM. The fasting glucose and 1 hr and 2 hr OGTT glucose cut-off values used for diagnosis.
(DOCX)
