# Supplementary material for: Adiponectin DNA methylation in South African women with gestational diabetes mellitus: Effects of HIV infection
Source: PLoS One. 2021 Mar 22;16(3):e0248694. doi: 10.1371/journal.pone.0248694 (PMC7984613; doi:10.1371/journal.pone.0248694)
Supplement: S1 Questionnaire — (DOCX) [file pone.0248694.s001.docx]

| **DATA COLLECTION SHEET**  **STUDY NUMBER**  **_______________**  ***AT RECRUITMENT:*** |
| --- |

1. **Date:
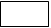

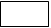
 /
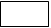

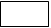
 / 2 0 1
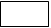
**
2. **Participant’s Name: ______________________________________________**
3. **Participant’s ID Number:
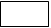

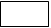

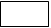

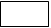

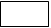

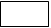

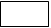

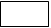

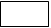

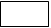

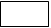

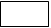

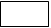
**
4. **Telephone Number:
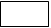

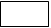

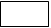

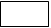

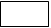

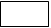

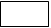

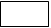

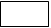

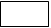
**

**
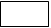

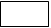

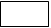

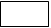

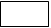

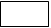

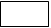

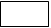

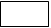

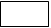
**

1. **Address: _______________________________________________________**

**________________________________________________________________**

1. **Age:
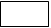

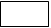
 years**
2. **Ethnicity: Black South African
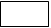
 Indian
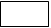
 Coloured
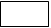
**

**White
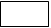
 Other
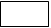
 _______________________________**

1. **Education – highest grade passed? __________________________________**
2. **Employment: Unemployed
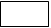
**

**Employed
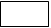
 . . . . Formally
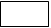
 Informally
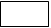
**

**Receives grants
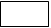
 Type of grant _____________________**

1. **Measure of socio-economic status^42^:**

**Housing quality index please circle correct number**

| ***Variables*** | ***Values*** | | |
| --- | --- | --- | --- |
| **Wall** | 0 = cardboard/plastic bags | 1 = metallic sheets (zinc), boards, wood | 2 = masonry (bricks, cement blocks) |
| **Floor** | 0 = dirt, cardboard, plastic bags | 1 = cement, tiles, brick, wood |  |
| **Roof** | 0 = cardboard, plastic bags | 1 = metallic sheet, wood, asbestos | 2 = tiles, cement, brick |
| **Electricity** | 0 = no | 1 = yes |  |
| **Water supply** | 0 = piped in street | 1 = piped in yard | 2 = piped indoors |
| **Sanitation** | 0 = in street, neighbour | 1 = in yard | 2 = inside house |
| **Type of sanitation** | 0 = homemade pit latrine | 1 = non-flush septic tank | 2 = flush |

1. **Parity
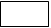

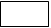
 Gravidity
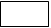

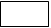
**
2. **Last Normal Menstrual period:
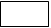

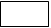
 /
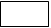

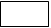
 / 201
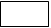
**
3. **Gestational age:
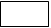

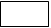
weeks
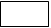
 days**
4. **Symphysis Fundal Height:
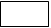

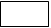
 cm**
5. **Height
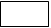

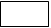

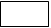
 cm Weight
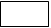

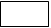

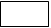
 kg**

**MUAC
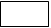

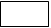
 cm**

1. **Blood Pressure
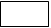

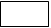

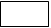
 /
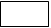

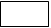

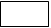
 mmHg**
2. **Haemoglobin
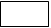

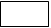
 .
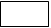
 g/dL**
3. **Urine dipstick: Protein
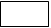
Glucose
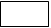
Blood
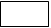
Ketones
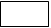
**
4. **Acanthosis nigricans: Yes
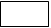
 No
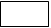
**
5. **Family History of Diabetes Mellitus: Yes
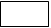
 No
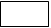
**

**If yes, relationship: _______________________________________________**

1. **Previous stillbirth or baby with congenital abnormality:**

**Yes
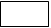
 No
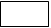
**

**If yes, details: ____________________________________________________**

1. **Previous baby ≥4kg: Yes
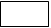
 No
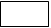
**
2. **Gestational Diabetes in prior pregnancy: Yes
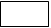
 No
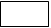
**
3. **History of Polycystic Ovarian Syndrome: Yes
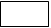
 No
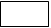
**
4. **HIV Status:
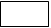
Negative
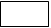
Positive
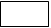
Unknown**
5. **Is the patient on antiretroviral. If so, which drugs:**

**_________________________________________________________________**

1. **Other medical conditions: __________________________________________**
2. **Drug history: ____________________________________________________**
3. **Random Glucose:**

**Glucometer (venous) g/dL**

**Glucometer (capillary) g/dL**

**Laboratory g/dL**

1. **HbA1C:**

**On-Site Test g/% Laboratory g/%**

***2-WEEKS LATER:***

1. ***Fasting glucose – 2 weeks later:***

**Date: / / 2 0 1**

**Gestational age: weeks days**

**Glucometer (venous) g/dL**

**Glucometer (capillary) g/dL**

**Laboratory g/dL**

***24-28 WEEKS PREGNANT:***

1. ***OGTT and repeat HbA1C – 24-28 weeks pregnant:***

**Date: / / 2 0 1**

**Gestational age: weeks days**

**HbA1C On-Site Test g/% HbA1C Lab g/%**

**OGTT – Glucometer (venous):**

**0hour 1 hour 2 hour**

**OGTT – Glucometer (capillary):**

**0hour 1 hour 2 hour**

**OGTT - Laboratory: 0hour 1 hour 2 hour**
