# Supplementary material for: Adiponectin DNA methylation in South African women with gestational diabetes mellitus: Effects of HIV infection
Source: PLoS One. 2021 Mar 22;16(3):e0248694. doi: 10.1371/journal.pone.0248694 (PMC7984613; doi:10.1371/journal.pone.0248694)
Supplement: S1 Fig — The percentage (%) of methylation as determine (y-axis) for a) 4 CpGs (-3412, -3410, -3400, -3372) in R1, b) 2 CpGs (-473, -415) in R2 and c) 2 CpGs (-112, -45) in R3. (DOCX) [file pone.0248694.s002.docx]

a)

b)

c)

**S1 Fig. Primer sensitivity using known methylated standards (x-axis) for each pyrosequencing probe.** The percentage (%) of methylation as determine (y-axis) for a) 4 CpGs (-3413, -3410, -3400, -3372) in R1, b) 2 CpGs (-473, -415) in R2 and c) 2 CpGs (-112, -45) in R3.
