# Supplementary material for: Adiponectin DNA methylation in South African women with gestational diabetes mellitus: Effects of HIV infection
Source: PLoS One. 2021 Mar 22;16(3):e0248694. doi: 10.1371/journal.pone.0248694 (PMC7984613; doi:10.1371/journal.pone.0248694)
Supplement: S2 Fig — DNA methylation levels at CpG -3413, -3410, -3400, -3372 and -415. *p<0.05. (DOCX) [file pone.0248694.s003.docx]

**S2 Fig.** **DNA methylation levels in HIV positive women receiving antiretroviral therapy (ART) (n=36) and those who were ART naïve (n=69).** DNA methylation levels CpG -3413, -3410, -3400, -3372, -415 and -112. ^*^p<0.05.
