# Supplementary material for: Adiponectin DNA methylation in South African women with gestational diabetes mellitus: Effects of HIV infection
Source: PLoS One. 2021 Mar 22;16(3):e0248694. doi: 10.1371/journal.pone.0248694 (PMC7984613; doi:10.1371/journal.pone.0248694)
Supplement: S1 Table — The target sequence, chromosomal location, target sequence and amplicon length used to design CpG assays. (DOCX) [file pone.0248694.s004.docx]

**S1 Table. Primer design for CpG sites in *ADIPOQ.*** The target sequence, chromosomal location, target sequence and amplicon length used to design CpG assays.

| **Name** | **Target sequence*** | **Chromosomal region** | **PCR and pyrosequencing primer*** | **Target sequence after NaBis treatment*** | **Amplicon length** |
| --- | --- | --- | --- | --- | --- |
| Assay 1 | **CGCG**GTGGCTCA**CG**CCTGTCATTCCAGCACTTTGGGAGGC**CG** | Chromosome 3:  186,839,297-186,839,338  Positive strand | Fwd1: GGTGGTAGGAGGTGATAGTTTAA  Rev1: ACTCCCCACCTCAAATAATCCAC  Seq1: GAAATGTTTTTTTGGTTAGG | YGYGGTGGTTTAYGTTTGTTATTTTAGTATTTTGGGAGGTYGAGGGGGTGGAT TATTTGAGGT | 199 (4CpGs) |
| Assay 2 | CTGAA**CG**TACACAGTCTCAGACTTAATCATGCACAGTGAGCAAGACTGTGGTGTGATAATTGG**CG**TCCCTGAC | Chromosome 3:  186,842,231-186,842,303  Positive strand | Fwd3: TTAGGTTAGAGAGTGGAGGATGTG  Rev3: TCCCCCTCCCATAAATTTACC  Seq3: CTCCCATAAATTTACCCTAATA | AATCAAAAACRCCAATTATCACACCACAATCTTACTCACTATACATAATTAAATCTAAAACTATATACRTTCAAACAATAAATACTTCAAAAAAAAACA | 186 (2CpGs) |
| Assay 3 | TTTGTTTAT**CG**GTTTTTGGTTTTTATTGAGTTGGTTAATGGGAAATGATAATTGTGAGGTGGGGATTGTTTGTTTT**CG**TGAG | Chromosome 3:  186,842,599-186,842,672  Positive strand | Fwd2: GTGGGTAATTGTTAGGGATATGT  Rev2: AAAAAATAACCCAACCTCAACAAC  Seq2: GTAATTGTTAGGGATATGTG | TTTGTTTATYGGTTTTTGGTTTTTATTGAGTTGGTTAATGGGAAATGATAATTGTGAGGTGGGGATTGTTTGTTTTYGTGAGTATTAGGTTGTTGAGGTTGGGTTAT | 136 (2CpGs) |

*5’ to 3’ orientation; NaBis - Sodium Bisulfite; Red bold type letters indicated CpG sites investigated on the adiponectin gene (ADIPOQ) promoter region.
