# Supplementary material for: Adiponectin DNA methylation in South African women with gestational diabetes mellitus: Effects of HIV infection
Source: PLoS One. 2021 Mar 22;16(3):e0248694. doi: 10.1371/journal.pone.0248694 (PMC7984613; doi:10.1371/journal.pone.0248694)
Supplement: S2 Table — The fasting glucose and 1 hr and 2 hr OGTT glucose cut-off values used for diagnosis. (DOCX) [file pone.0248694.s005.docx]

**S2 Table. Analysis of International Association of Diabetes in Pregnancy Study Group criteria in diagnosing GDM.** The fasting glucose and 1 hr and 2 hr OGTT glucose cut-off values used for diagnosis.

| **Abnormal glucose readings** | **Women diagnosed N (%)** |
| --- | --- |
| Fasting glucose alone (≥ 5.1 mmol/L) | 80 (84.2 %) |
| 1 hr glucose alone (≥ 10.0 mmol/L) | 0 (0 %) |
| 2 hr glucose alone (≥ 8.5 mmol/L) | 2 (2.1 %) |
| Fasting glucose + 1 hr + 2 hr glucose | 2 (2.1 %) |
| Fasting glucose + 1 hr glucose | 1 (1.1 %) |
| Fasting glucose + 2 hr glucose | 10 (10.5 %) |
| 1 hr glucose + 2 hr glucose | 0 (0 %) |
| Total number of participants with GDM | 95 (100 %) |

HIV^-^ GDM^+^ (n=63); HIV^+^GDM^+^ (n=32)
